# Regulation rewiring analysis reveals mutual regulation between STAT1 and miR-155-5p in tumor immunosurveillance in seven major cancers

**DOI:** 10.1038/srep12063

**Published:** 2015-07-09

**Authors:** Chen-Ching Lin, Wei Jiang, Ramkrishna Mitra, Feixiong Cheng, Hui Yu, Zhongming Zhao

**Affiliations:** 1 Department of Biomedical Informatics, Vanderbilt University School of Medicine, Nashville, Tennessee 37203, USA; 2 Department of Cancer Biology, Vanderbilt University School of Medicine, Nashville, Tennessee 37203, USA; 3 Department of Psychiatry, Vanderbilt University School of Medicine, Nashville, Tennessee 37203, USA

## Abstract

Transcription factors (TFs) and microRNAs (miRNAs) form a gene regulatory network (GRN) at the transcriptional and post-transcriptional level in living cells. However, this network has not been well characterized, especially in regards to the mutual regulations between TFs and miRNAs in cancers. In this study, we collected those regulations inferred by ChIP-Seq or CLIP-Seq to construct the GRN formed by TFs, miRNAs, and target genes. To increase the reliability of the proposed network and examine the regulation activity of TFs and miRNAs, we further incorporated the mRNA and miRNA expression profiles in seven cancer types using The Cancer Genome Atlas data. We observed that regulation rewiring was prevalent during tumorigenesis and found that the rewired regulatory feedback loops formed by TFs and miRNAs were highly associated with cancer. Interestingly, we identified one regulatory feedback loop between *STAT1* and miR-155-5p that is consistently activated in all seven cancer types with its function to regulate tumor-related biological processes. Our results provide insights on the losing equilibrium of the regulatory feedback loop between *STAT1* and miR-155-5p influencing tumorigenesis.

Transcription factors (TFs) are proteins that bind to specific nucleic acid sequences to regulate target gene expressions at the transcriptional level^1^. MicroRNAs (miRNAs) are small (∼21–22 nucleotides), noncoding RNAs that regulate gene expression at the post-transcriptional level in eukaryotic cells^2^. TFs and miRNAs are the two major families of gene regulators that constitute global regulatory networks (GRNs) in metazoans^3^. Like other biological networks, TF and miRNA regulatory networks are scale-free, that is, a few nodes in the networks are highly connected^4^. However, experimentally validated regulatory networks may possess literature bias^5^, whereas predicted ones tend to be noisier because of a high false-positive rate of predicted regulatory relationships. Expression profiles have been widely applied to regulatory networks in order to either filter out the potential false-positive regulations or predict the context-specific activity of regulations^6^. In other words, the application of expression profiles could facilitate the discovery of dynamic regulatory networks.

TFs can be either activators or repressors^7^. They are generally regarded as the primary regulators of gene expression^8^, whereas miRNAs usually suppress target mRNA expression^9^. Of note, miRNAs are also considered critical in regulatory systems due to their ability to fine-tune the expression of a target gene^10^,^11^. Accordingly, the co-operations of TFs and miRNAs may be subtle. Previous studies have observed that co-regulations between TFs and miRNAs are prevalent within living cells^3^. Therefore, it is important to investigate TF-miRNA co-regulations, and some studies have discovered interesting results^12^,^13^,^14^. Among the diverse co-regulatory relationships between TFs and miRNAs, the feed-forward loops (FFLs) and feedback loops (FBLs) receive the most intensive research attention^3^. While TF-miRNA FFLs can function in noise buffering, FBLs are pivotal in controlling cell cycle progression through auto-regulation^15^,^16^. Even though much progress has been made, co-regulations between TFs and/or miRNAs remain elusive to a large extent.

To address this knowledge gap, we constructed a GRN that constituted the TF and miRNA regulations derived from ChIP-Seq and CLIP-Seq data, respectively. In order to investigate the regulation activity and rewiring, we further integrated the GRN using mRNA and miRNA expression profiles in seven major cancer types generated from The Cancer Genome Atlas (TCGA). Through our analyses, we predicted an important FBL between *STAT1* and miR-155-5p — the most differentially co-expressed regulator pair between normal and tumor samples. Furthermore, literature-based evidence supported our network-based results and indicated that the disequilibrium of this FBL may affect tumor immunosurveillance. In summary, our study clarified the regulation dynamics of TF and miRNA through network and rewiring in multiple types of cancer as well as unveiled the critical role of mutual regulations between TF and miRNA in tumorigenesis.

## Results and Discussion

### TF and miRNA regulatory activity in cancers

We obtained TF and miRNA regulation data to construct human gene regulatory networks (GRN) at both the transcriptional and post-transcriptional level (See Methods and Supporting Information S1). Through the use of preliminary analyses, we observed that the predicted GRN may possess a false positive rate, while the experimentally validated GRN may be subject to literature bias (Supporting Information S2). Additionally, we found that by incorporating the expression correlation constraint between regulators and targets, we could control the false positive rate of the predicted GRN and reduce the literature bias of the experimentally validated GRN (Supporting Information S2). Moreover, previous studies have demonstrated that an integration of expression correlation to GRN could facilitate retrieving the highly confident regulations^17^,^18^. Aside from these advantages, the application of expression correlations between regulators and targets facilitates the identification of the condition-specific GRN for each cancer type. Therefore, we used the correlated GRN to perform the following analyses.

**Figure 1:**
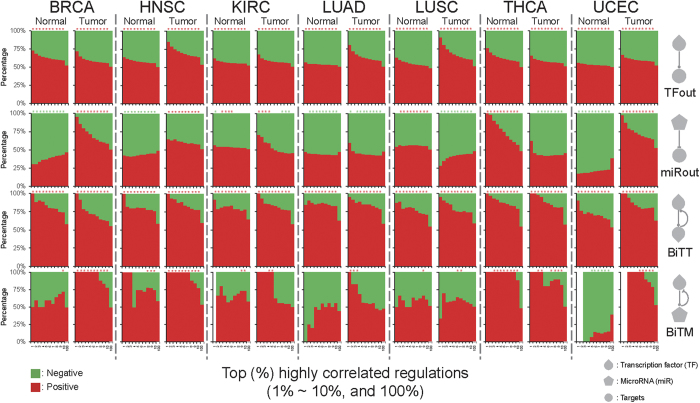
Regulatory activity of TF and miRNA in cancers. The correlation patterns of four regulation types across the seven TCGA cancer types. The regulation types are labeled on the right-hand side. For each cancer type, the percentage of positive and negative regulations is shown as a function of the top 1% to 10% and 100% correlated GRNs. In each cancer type, the left and right sub-column represents the correlated GRN derived from normal and tumor samples, respectively. The asterisk shows the enrichment significance of positive or negative regulations with *P* < 0.05, as produced by Fisher′s exact test. The red (green) asterisks indicate that the positive (negative) regulations are significantly overrepresented.

To impart regulation activity, we considered expression correlations between regulators and target genes. A positive or negative correlation hints to a potential activation or repression mediated by a regulator, respectively. We first categorized regulations into four types: 1) unidirectional TF to target (TFout), 2) unidirectional miRNA to target (miRout), 3) bidirectional TF to TF (BiTT), and 4) bidirectional TF to miRNA (BiTM). We observed that positive correlations are more prevalent than negative ones in TFout and BiTT regulations over all seven cancer types (Fig. 1, TFout and BiTT). Moreover, the proportions of positively correlated BiTT regulations are even larger than negative ones when compared to TFout regulations (Fig. 1, BiTT). Observations in the surveyed cancerous and paracancerous tissues suggest that activating regulations might prevail over TF regulation. Furthermore, they also imply that regulatory circuits between two TFs may mutually induce expression levels in each other. Unlike in TF regulations, no universal pan-cancer regulatory pattern was found in miRNA regulation (Fig. 1, miRout). This result is in accordance with the notion that miRNAs do not dominate gene expression regulation, and their regulations are likely disturbed by other regulators in cancers^3^,^15^,^19^. As stated earlier, miRNAs have demonstrated their ability to fine-tune target gene expression in order to regulate molecular mechanisms in cells^10^,^11^. In this way, miRNA regulations are critical to living cells, even if they might not dominate the regulation of target gene expression. Another relevant assumption is that miRNA regulation activity is easily affected by other co-regulators, i.e., context-dependent regulation activity^3^,^15^. On the other hand, like TFout and BiTT regulations, the positively correlated BiTM regulations are more frequently observed than the negatively correlated ones in most cases (Fig. 1, BiTM). Combined with the above investigations, the stronger positive regulation activity of BiTM may be attributed to the positive and dominant regulatory activity of TF and the fine-tuning regulation of miRNAs.

### Regulation rewiring during tumorigenesis

**Figure 2:**
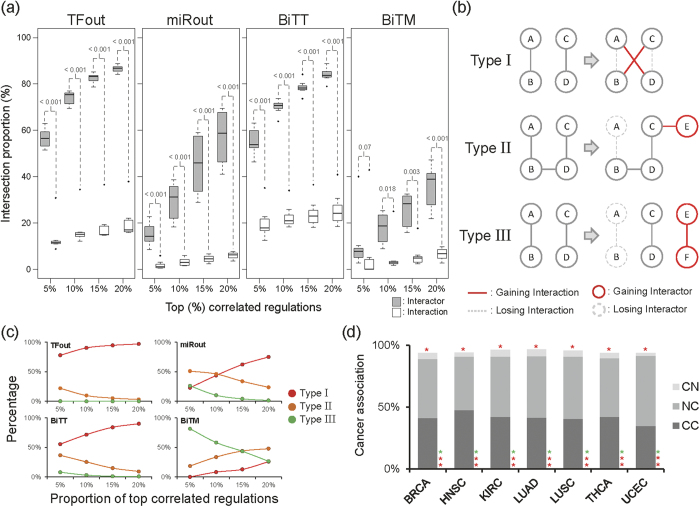
Regulation rewiring in cancers. (**a**) The patterns of joint interactors/interactions between normal and tumor GRNs. For each regulation type, the proportions of the intersection regulators/regulations between normal and tumor GRNs across seven cancer types are shown as a function of the proportion of the top correlated regulations. The *P*-values are calculated by Wilcoxon rank-sum test to indicate the significance of the difference between the proportions of shared interactors and interactions. (**b**) The illustration of regulation rewiring. We defined three types of regulation rewiring: I) gain or loss of a regulation but retention of both the regulator and target, II) gain or loss of a regulation with either one regulator or target being kept, and III) gain or loss of a regulation through the gain or loss of both the regulators and targets. (**c**) The distribution of regulation rewiring. For each regulation forms, the average percentage of regulation rewiring types over seven cancer types is shown as a function of the top 5%, 10%, 15, and 20% highly correlated GRNs. The regulation rewiring types are displayed by color codes (red: Type I, orange: Type II, and green: Type III). (**d**) The enrichment of cancer-associated regulations within DC BiTM regulations in the seven TCGA cancer types. For each cancer type, the proportion of cancer-associated regulations is shown. The asterisk on the top of each bar represents the significance of cancer-associated regulations with *P* < 0.05 derived from Fisher′s exact test. Because BiTM regulations possess two types of regulators, i.e., TF and miRNAs, we further specialized these three categories for BiTM as: 1) CN: only TFs are cancer-associated; 2) NC: only miRNAs are cancer-associated; 3) CC: both TFs and miRNAs are cancer-associated (C: cancer-associated, N: non-cancer-associated). In addition, we labeled the significance of the three sub-categorized cancer-associated regulations with *P* < 0.05 from Fisher’s exact test on the bottom of each bar. The order of asterisks for the sub-categorized cancer-associated regulations is CN, NC, and CC from top to bottom. Red asterisk: overrepresented. Green asterisk: underrepresented.

To further explore the characteristics of regulation activity in tumorigenesis, we examined the variability of the regulation correlation between tumor and normal samples in seven studied cancer types. Herein, we considered the top 5%, 10%, 15%, and 20% correlated regulations as four putative data sets showing biologically meaningful regulatory activity within cellular systems. We observed that the proportion of overlapped interactors (regulators and targets) between normal and tumor samples are significantly larger than those of overlapped interactions (regulations), except for the top 5% correlated BiTM (Fig. 2a). This above investigation may uncover that the regulations were rewired during cancer development. Regulation rewiring indicates that changes occurring in regulation between regulators and targets as conditions switched, which has been found to be crucial in gaining or losing biological functions during evolution^20^,^21^. Moreover, the differential co-expression has been shown to be capable of identifying the dysfunctional regulatory relationships in diseases^22^. Importantly, this observation showed that regulation rewiring is persistent across seven studied cancer types and therefore suggested that regulation rewiring might be a common mechanism during tumorigenesis (Fig. 2a). Herein, we further categorized regulation rewiring into three types (Fig. 2b). For type I regulation rewiring, the proportion of the shared interactors is large but small for shared interactions. For type III, both the proportions of the shared interactors and interactions are small. Type II has intermediate extent comparing to types I and II in terms of the proportions of shared interactors and interactions.

We observed that type I regulation rewiring is dominant in TFout and BiTT over the four criteria of top correlated regulations, i.e. top 5%, 10%, 15%, and 20% (Fig. 2c, two left panels). Interestingly, the miRNA-involved regulations, miRout and BiTM, exhibit distinct patterns from the TF-involved ones, TFout and BiTT (Fig. 2c, two right panels). miRout shows the prevalence regarding regulation rewiring of type II in the top 5% and type I in the top 15% and 20% highly correlated regulations, respectively (Fig. 2c). Notably, type III regulation rewiring prevails over the top 5% and 10% of highly correlated BiTM regulations (Fig. 2c). This result implies that highly correlated BiTM regulations might result in a complete loss or gain during tumorigenesis. In other words, the gain or loss of BiTM regulations with biologically meaningful regulatory activity may be associated with cancer development.

To probe the importance of BiTM regulation rewiring in cancer, we investigated differentially correlated (DC) regulations between normal and tumor samples. The DC regulations were defined by the distance of Fisher transformed Spearman′s ρ between normal and tumor (see Methods in detail). We further collected cancer-associated genes and cancer-associated miRNAs from public databases and literatures (see Methods for detail) and defined the cancer-associated regulations, i.e., regulations formed by cancer-associated genes or miRNAs, to interrogate the association between DC BiTM regulations and cancer. Indeed, we found that the cancer-associated regulations were significantly overrepresented in DC BiTM regulations across seven studied cancer types (Fig. 2d, *P* < 0.05, Fisher’s exact test). The proportion of cancer-associated regulations in DC BiTM regulations was on average approximately 95% in seven studied cancer types. This observation strongly suggests that DC BiTM regulations might be crucial to cancer development. Additionally, regulations composed of cancer-associated TFs and non-cancer-associated miRNAs are significantly infrequent in DC BiTM regulations compared to background GRN (Fig. 2d, *P* < 0.05, Fisher’s exact test, category CN). Notably, the cancer-associated TFs used in this study are integrally involved in cancers through mutation^23^,^24^. A reasonable scenario is that these cancer-associated TFs are involved in cancer development via their mutations rather than through differential regulatory activity. On the other hand, those regulations comprised of non-cancer-associated TFs and cancer-associated miRNAs are significantly overrepresented in DC BiTM regulations (Fig. 2d, *P* < 0.05, Fisher’s exact test, category NC). This observation suggests that these non-cancer-associated TFs might be implicated in cancer development through the possession of differential regulatory activity in the regulation of cancer-associated miRNAs instead of mutations. Interestingly, the regulations comprised of cancer-associated TFs and cancer-associated miRNAs are prevalent in DC BiTM regulations (Fig. 2d, *P* < 0.05, Fisher’s exact test, category CC). This observation further indicates that the mutations of these cancer-associated TFs might also influence their regulatory activity in cancer development. The combination of the latter two results demonstrates that DC feedback regulations of cancer-associated miRNAs on TFs (both cancer-associated and non-cancer-associated) might play a crucial role in cancer. Briefly, the aforementioned results indicate that mutual regulations between miRNAs and TFs might be critical to tumorigenesis. Besides, some significant patterns of cancer association were also observed in three other types of regulations, i.e., TFout, miRout, and BiTT (Supporting Information S3). In summary, the abovementioned results further propose that regulation rewiring may play a pivotal role in cancer development.

### Identification of the negative feedback regulatory loop between STAT1 and miR-155-5p

**Figure 3:**
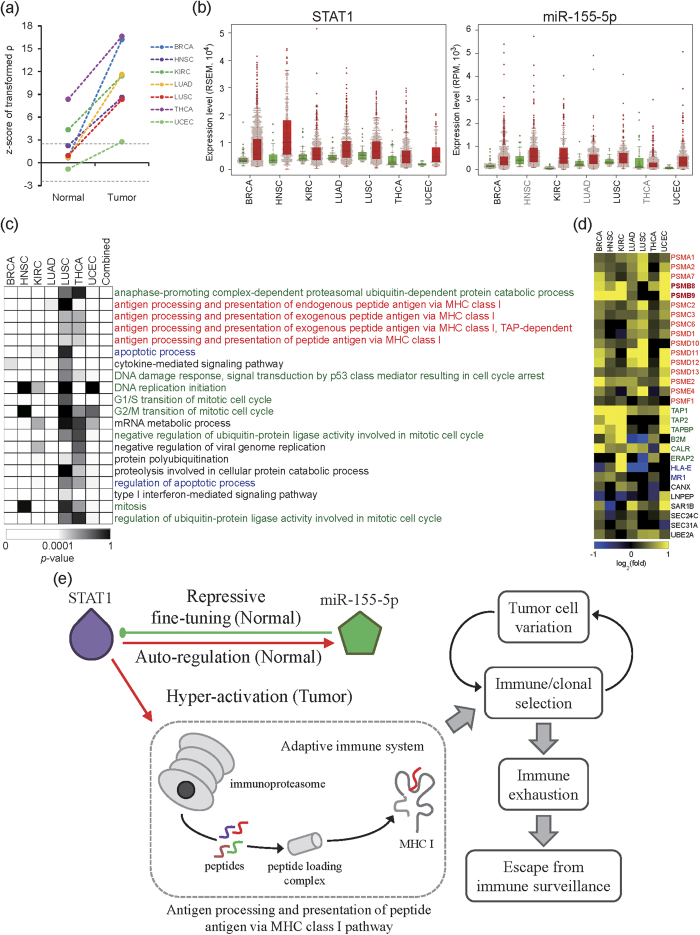
The auto-regulation between STAT1 and miR-155-5p. (**a**) Differential co-expression of the BiTM regulations formed by STAT1 and miR-155-5p. For each cancer type, the z-score of the transformed ρ of the auto-regulation formed by STAT1 and miR-155-5p in normal and tumor samples is shown. The two dash lines show a z-score of -2.5 and 2.5, respectively. The significance of |z-score| > 2.5 corresponds to *P* < 0.01. (**b**) The expression profiles of *STAT1* and miR-155-5p in normal and tumor samples across the seven TCGA cancer types. The cancer type labeled in black indicates that the difference in expression profiles between tumor and normal is significant (*P* < 0.05, by edgeR). Green box: normal; red box: tumor. (**c**) The top 20 significantly enriched regulatory functional modules of *STAT1*. The GO functions colored by green, blue, and red display mitosis, apoptosis, and major histocompatibility complex (MHC) class I related functional modules, respectively. The column labeled with ″Combined″ represents the combined *P*-value of seven cancer types by Fisher′s method. For each cancer type, the *P*-value of significance for each functional module is indicated by the color code below the heat map. (**d**) The log_2_ fold ratio of STAT1 target genes in MHC I related functions. The log_2_ fold ratio is calculated by edgeR (tumor vs. normal). For each cancer type, the log_2_ fold ratio of each STAT1 target gene is indicated by the color code below the heat map. (**e**) The proposed hypothesis of the downstream effects caused by the loss of equilibrium mediated by the STAT1-miR-155-5p feedback loop. The potential molecular mechanism describes how the disequilibrium of the regulatory feedback loop between *STAT1* and miR-155-5p triggers cancer immunoediting to escape from immunosurveillance.

To predict the putative BiTM regulations associated with tumorigenesis, we ranked all BiTM regulations according to their average distance of Fisher transformed Spearman′s ρ between normal and tumor samples across the seven studied cancer types. That is, we identified the highly DC BiTM regulations. We found that STAT1–miR-155-5p ranked the first in DC BiTM regulations across seven cancer types. That is, STAT1 and miR-155-5p are highly differentially co-expressed in all the seven cancer types. In normal samples, the expression of *STAT1* is significantly and positively correlated with that of miR-155-5p in clear cell kidney carcinoma (KIRC) and papillary thyroid carcinoma (THCA) (|z-score of transformed ρ| > 2.5), but not in breast cancer (BRCA), head and neck squamous cell carcinoma (HNSC), lung adenocarcinoma (LUAD), lung squamous cell carcinoma (LUSC), or uterine corpus endometrial carcinoma (UCEC) (Fig. 3a). Moreover, except for UCEC, the correlations between *STAT1* and miR-155-5p in normal samples are positive but not always significant (Fig. 3a). In all tumor samples, the expression profiles are significantly and positively correlated with each other (z-score of transformed ρ > 2.5) (Fig. 3a). The regulation of STAT1 on miR-155 expression was supported by two proposed STAT1 binding sites in the miR-155 promoter^25^. In addition, that study performed the electrophoretic mobility shift assay and confirmed the binding ability of STAT1 in the miR-155 promoter. These lines of evidence supported the notion that STAT1 could potentially activate miR-155 expression. Furthermore, the miR-155-mediated repression of *STAT1* expression has been demonstrated through the overexpression of miR-155 in cell lines^26^ and in miR-155-deficient mice^27^. Moreover, Selbach *et al*. demonstrated the down-regulation of STAT1 by over-expressing in the time series^28^. Accordingly, we hypothesized that mutual regulation between *STAT1* and miR-155-5p is a negative feedback loop: STAT1 activates miR-155, and miR-155 represses gene *STAT1*. Therefore, we observed neutral but positive correlations between *STAT1* and miR-155-5p in normal samples. This suggested a molecular regulation mechanism that STAT1 dominated this feedback loop, while miR-155 negatively fine-tuned the expression of *STAT1* in normal samples. Intriguingly, *STAT1* is significantly up-regulated in the seven studied cancer types (Fig. 3b, left panel, *P* < 0.05 by edgeR); miR-155-5p is also up-regulated in seven cancer types, but only significant in BRCA, KIRC, LUSC, and UCEC (Fig. 3b, right panel). That is, STAT1 was overexpressed in tumor samples. This overexpression also activated the miR-155-5p expression. As we mentioned, *STAT1* significantly and positively correlated with miR-155-5p in tumor samples across the seven studied cancer types. However, because this regulatory feedback loop was dominated by STAT1, the overexpression of *STAT1* might be stronger than the miR-155-5p-mediated regulation of STAT1 and, therefore, may have abated the repressive fine-tuning of miR-155-5p for *STAT1* expression. In summary, the identified STAT1-miR-155-5p feedback loop was supported by multiple lines of evidence and observed in all the seven cancer types we studied. Nevertheless, our approach for identifying regulatory motifs like the STAT1-miR-155-5p feedback loop is primarily computational, specific experimental work is warranted to further validate our results in future.

### STAT1-miR-155-5p feedback loop drives tumorigenesis

To investigate if losing the equilibrium controlled by the feedback loop of STAT1 and miR-155-5p is involved in tumorigenesis, we explored the STAT1 regulatory functional modules in tumor samples (Supporting Information S4)^29^,^30^. We collected the putative STAT1 target genes, which are significantly and positively correlated with STAT1 expression (Fisher transformed Spearman′s ρ > 2.5) and up-regulated in tumor samples (*P* < 0.05 by edgeR)^31^. That is, the selected target genes could be activated by STAT1 in a tumor. We then performed functional enrichment analysis for these activated target genes to identify STAT1 regulatory functional modules, which are activated in a tumor (see Methods in detail). According to the combined *p*-value derived from Fisher’s combined test across seven cancer types, we listed the top 20 significantly enriched STAT1-regulated functional modules in Fig. 3c. There are three major functional groups of STAT1-regulated modules: 1) mitosis, 2) apoptosis, and 3) major histocompatibility complex (MHC) class I related. Mitosis and apoptosis have been well-studied in cancers^32^,^33^, so we focused on the MHC class I related functional modules. The MHC class I is responsible for antigen processing and presentation to help CD8 T cells (cytotoxic T cells) recognize unhealthy cells, e.g. virus-infected cells or tumor cells. However, this process has dual roles in inhibiting tumorigenesis through cancer immune surveillance and cancer immunoediting, which facilitates a tumor escape from immunosurveillance^34^,^35^. Additionally, we found that genes involved in the STAT1 regulatory MHC class I related functional modules are highly expressed in the seven cancer types (Fig. 3d). These genes were further mapped to three major components of the antigen processing and presentation pathway: 1) proteasome, 2) peptide loading complex, and 3) MHC class I. The proteasome subunits were the most up-regulated in the modules across seven cancer types. Notably, there are two proteasome subunits, PSMB8 (also known as LMP7) and PSMB9 (also known as LMP2), included in the functional module. The proteasome that incorporates these two subunits is specialized as an immunoproteasome^36^,^37^. Furthermore, one of these two subunits, PSMB9, was reportedly regulated by STAT1^38^,^39^. The above results suggest that the overexpression of *STAT1* in tumor may highly activate the immunoproteasome and then trigger the downstream pathway, i.e., antigen processing and the presentation of a peptide antigen via the MHC class I pathway.

Based on the results above, we proposed a hypothesis on how the losing equilibrium of the regulatory feedback loop between *STAT1* and miR-155-5p influences tumorigenesis (Fig. 3e). In healthy (normal) cells, miR-155-5p auto-regulates *STAT1* by negatively fine-tuning *STAT1* expression^25^,^26^. However, in tumor cells, *STAT1* was overexpressed. This overexpression might interrupt the equilibrium of the feedback loop and further hyper-activate the immunoproteasome^38^,^39^, as well as possibly the downstream antigen processing and presentation pathway in which MHC class I is involved. The activation of antigen processing and presentation pathway brought tumor cells into the first phase of cancer immunoediting: elimination^34^,^35^. During tumorigenesis, tumor cells that are genetically unstable and mutated promptly produce abundant variants. Under immune selection pressure, many tumor cells were destroyed, but new tumor variants with distinct mutations that increased tumor immune resistance could survive. Darwinian selection process moved tumor cells into the second phase of cancer immunoediting: equilibrium^34^,^35^. Then, the survival of tumor cells that possessed insensitivity to immunologic detection and/or elimination caused immune exhaustion. Finally, tumor cells were able to escape from immune surveillance in the third phase of immunoediting: escape^34^,^35^. Collectively, our findings suggest that the disequilibrium of the regulatory feedback loop between *STAT1* and miR-155-5p might trigger cancer immunoediting in order to allow tumor cells to escape from immunosurveillance and even to promote tumorigenesis. However, more experiments are needed to validate our hypothesis.

### Identifying existing drugs potentially associated with *STAT1* in proteasome pathway

**Figure 4:**
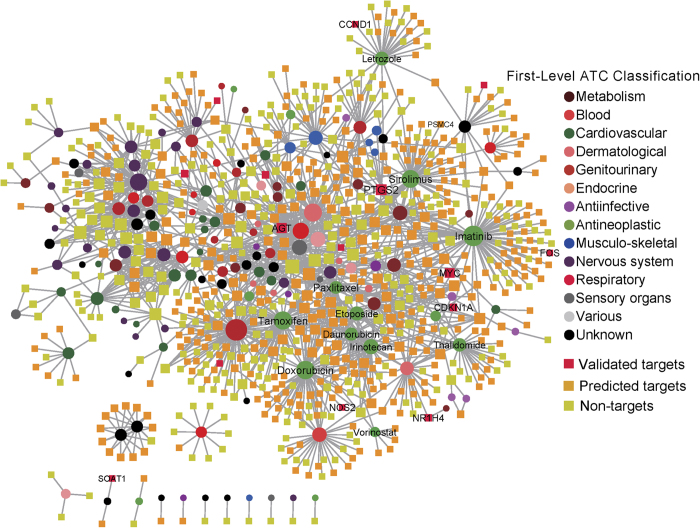
The *STAT1*-related drug-gene interaction subnetwork. This network contains 761 drug target genes (square) and 148 drugs (circle) that either down-regulate or up-regulate *STAT1* expression. The edges denote the drug-target interactions collected from DrugBank (v3.0), the Therapeutic Target Database (TTD), and the PharmGKB database. Genes were color-coded based on the *STAT1* experimentally validated targeted genes (validated targets, red squares), computationally predicted *STAT1* target genes (predicted targets, yellow squares), and unknown *STAT1* target genes (non-targets, cyan squares). All drugs were grouped using the anatomical therapeutic chemical (ATC) classification system codes. This graph was visualized using Cytoscape v2.8.3 (http://www.cytoscape.org/).

Proteasome inhibitors have been studied in cancer treatment^40^,^41^,^42^, and our study showed that STAT1 might activate proteasome in cancer. These previous observations have implied that STAT1 might possess therapeutic potential in molecular cancer therapy. To further explore this potential utility, we constructed a *STAT1*-related drug-gene association subnetwork (Fig. 4). In Fig. 4, circle nodes represent drugs that either down-regulated or up-regulated *STAT1* expression collected from the Connectivity Map (CMap, build 02)^43^; square nodes represent the drug target genes collected from the DrugBank (v3.0)^44^, Therapeutic Target Database (TTD)^45^, and PharmGKB database^46^ (see details in the Methods section). Drug nodes were colored-coded using the anatomical therapeutic chemical classification system based on previous studies^47^. Genes were divided into three subgroups: *STAT1* experimentally validated target genes (red), *STAT1* computationally predicted target genes (yellow), and non-target genes (light green). As shown in Fig. 4, several antineoplastic drugs, including sirolimus, imatinib, thalidomide, tamoxifen, irinotecan, doxorubicin, paxlitaxel, letrozole, are connected to many *STAT1* experimentally validated or computationally predicted targeted genes. For example, sirolimus, a canonical allosteric inhibitor of the mTOR kinase with immunosuppressant and pro-apoptotic activities, possesses both antifungal and antineoplastic properties. A recent study found that *in vitro* sirolimus regulates the proteasome, such as the 26S protease regulator subunit 6B encoded by *PSMC4* gene, at low micromolar concentrations^48^. Thalidomide, an immunosuppressive and anti-angiogenic agent, inhibits the release of tumor necrosis factor-alpha from monocytesm and modulates other cytokine action. Hernandez Mde *et al*. found that thalidomide inhibits inflammation and nuclear factor-κB activity that is linked to the proteasome pathway^49^. Vorinostat, a pan-HDAC inhibitor, is used for the treatment of cutaneous T-cell lymphoma. A recent study showed that the proteasome inhibitor carfilzomib interacts synergistically with vorinostat in jurkat T-leukemia cells^50^. Two previous studies also found the synergy between proteasome inhibitors and imatinib mesylate in chronic myeloid leukemia^51^,^52^. Put together, our drug-gene network analysis could pinpoint the potential associations between STAT1-regualted target genes and FDA-approved or experimental drugs. It is worth noting that while CMap data is innovative and powerful, it has limitation on the lack of control for the selection of the optimal drug dose, which should be subtoxic to produce informative expression profile data. For example, compounds used at higher concentration are expected to lead to widespread effects due to drug off-target and secondary effect that are difficult to control in a high-throughput setting. Thus, our network analysis here is for exploratory purpose, and further experimental validation of predicted associations between STAT1-regualted target genes and existing drugs (Fig. 4) is warranted.

## Methods

### The TF-miRNA regulatory networks

We obtained the TF regulations from ChIPBase v1.1^53^, a resource collected transcriptional regulations decoded from ChIP-Seq data. Here, the regulatory regions were defined by default as upstream 5kb and downstream 1 kb. The miRNA regulations were extracted from starBase v2.0^54^. starBase collected miRNA regulations from CLIP-Seq data. In order to retrieve relatively reliable miRNA-mRNA interactions, we only adopted those interactions supported by at least 3 experiments in this study. We combined these two resources into the predicted GRN. The TF and gene names were also mapped to Entrez IDs, and all miRNAs were converted to mature miRNAs and mapped to miRBase v20 accession numbers^55^. As a result, the predicted GRN involves 825,659 regulations among 107 TFs, 1,851 miRNAs, and 18,705 target genes.

For the experimentally validated GRN, the TF regulations were obtained from TRANSFAC^®^ Professional database (Release: 2014.2)^56^, and the miRNA regulations were extracted from TarBase v6.0^57^ and TRANSFAC^®^ Professional database. In TarBase, we only reserved the miRNA regulations that had been validated by low-throughput experiments with reporter gene, northern blot, western blot, or qPCR. The regulations between TFs and miRNAs were obtained from TransmiR v1.2^58^ and TRANSFAC^®^ Professional database. We constructed the experimentally validated GRN by combining all of the above TF and miRNA regulations and eliminating all self-loops. Finally, the experimentally validated GRN consists of 10,046 regulations among 597 TFs, 497 miRNAs and 2,581 target genes.

### Cancer-associated genes and miRNAs

In this study, we compiled a mutated cancer-associated gene set by merging three large-scale cancer genome projects: 1) 125 mutated cancer genes from genome-wide sequencing studies of 3,284 tumors using the 20/20 rule^59^; 2) 127 significantly mutated genes from 3,281 tumors in 12 main cancer types^23^; 3) 487 experimentally validated and well-curated cancer genes from the Cancer Gene Census^24^ (July 10, 2013). Collectively, the union of these four gene sets, which covers 604 genes, provides a high-quality cancer gene set for the follow-up analysis.

We retrieved a total of 325 cancer-associated miRNAs from miRCancer database^60^ (updated in June 14^th^, 2014), a database that provides a comprehensive curation of differentially expressed miRNAs from published literature in PubMed. These 325 miRNAs were reportedly associated with 161 cancer types in the database.

### Identification of differentially co-expressed regulations

In this study, we applied Spearman′s ρ to assess the co-expression level between regulators and target genes. The mRNA and miRNA expression profiles of seven cancer types—breast cancer (BRCA), head and neck squamous cell carcinoma (HNSC), clear cell kidney carcinoma (KIRC), lung adenocarcinoma (LUAD), lung squamous cell carcinoma (LUSC), papillary thyroid carcinoma (THCA), and uterine corpus endometrial carcinoma (UCEC)—were downloaded from The Cancer Genome Atlas (TCGA) on 10/02/2013 (see Supporting Information S5 for details). To diminish the bias from sample number difference, Spearman′s ρ was further transformed by Fisher transformation and standardized as below:
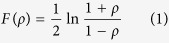

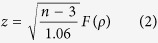
where *F*(*ρ*) is the function of Fisher transformation; *z* is a standard score of transformed *ρ*; and *n* is the number of matched samples between regulators and targets. We further denoted the paired regulator and target gene with |*z*| ≥ 2.5 (the corresponding significance is *P* < 0.01) as significantly correlated regulations. For each significantly correlated regulation, we calculated the difference of *z* between normal and tumor as below:
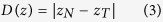
where *z*_*N*_ (*z*_*T*_) is the standard score of the studied regulation in normal (tumor) samples. Finally, we defined the significantly correlated regulations with top 10% *D*(*z*) as differentially co-expressed in the corresponding cancer type.

### Constructed STAT1-related drug-gene interaction subnetwork

We collected drugs that either down-regulated or up-regulated *STAT1* expression from the Connectivity Map (CMap, build 02)^43^. The CMap comprises over 7,000 gene expression profiles from cultured human cell lines that are treated by 1,309 bioactive small molecules across different concentrations, covering 6,100 individual instances. The CMap provides a measure of the extent of differential expression of a given probe set. Herein, amplitude *a* is defined as follows:
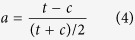
where t is the scaled and thresholded average difference value for the drug treatment group, and *c* is the thresholded average difference value for the control. In the above equation, *a* = 0 indicates no differential expression, *a* > 0 indicates increased expression (up-regulation) upon treatment, and *a* < 0 indicates decreased expression (down-regulation) upon treatment. In this study, we used the drug-STAT1 signatures that an amplitude value is more than 0.67 (more than two-fold) as up-regulation drug-gene pairs, and an amplitude value is less than −0.67 as down-regulation drug-gene pairs. We found 201 drugs that down-regulate STAT1 expression and 166 drugs that up-regulate *STAT1* expression (Supporting Information S6), using the above criteria.

We next mapped the drugs that are involved in *STAT1* down- or up-regulation into DrugBank database. In total, 171 FDA-approved or experimental drugs were found. We then collected the drug-gene interactions for those 171 FDA-approved or experimental drugs from three public databases: DrugBank (v3.0)^44^, Therapeutic Target Database (TTD)^45^, and PharmGKB database^46^. Drugs were grouped using the anatomical therapeutic chemical (ATC) classification system codes^61^. All genes were mapped and annotated using the gene Entrez IDs and official gene symbols based on NCBI database^62^. All duplicated drug-gene interaction pairs were removed. In total, we obtained 1,528 pairs connecting 148 drugs and 761 drug target genes. All network visualization and related network topological parameters were presented using Cytoscape v2.8.3^63^.

## Additional Information

**How to cite this article**: Lin, C.-C. *et al*. Regulation rewiring analysis reveals mutual regulation between STAT1 and miR-155-5p in tumor immunosurveillance in seven major cancers. *Sci. Rep*. **5**, 12063; doi: 10.1038/srep12063 (2015).

## Supplementary Material

Supplementary Information

Supplementary table S1
