# Supplementary material for: Regulation rewiring analysis reveals mutual regulation between STAT1 and miR-155-5p in tumor immunosurveillance in seven major cancers
Source: Sci Rep. 2015 Jul 9;5:12063. doi: 10.1038/srep12063 (PMC4496795; doi:10.1038/srep12063)
Supplement: Supplementary Information [file srep12063-s1.pdf]

# Supporting Information

# Regulation rewiring analysis reveals mutual regulation between STAT1 and miR-155-5p in tumor immunosurveillance in seven major cancers

Chen-Ching Lin, Wei Jiang, Ramkrishna Mitra, Feixiong Cheng, Hui Yu, Zhongming Zhao

## Contents

**S1 Descriptive statistics of the gene regulatory network structure** .......... 2
Table S1: Number of regulations in the GRNs .......... 2
**S2 Reliability of the gene regulatory network** .......... 3
Figure S1: Regulation degree distribution of the GRNs .......... 4
Figure S2: Out-degree distribution profile of correlated GRNs .......... 4
Table S2: Correlation between out-degree and the number of publications of regulators .......... 5
**S3 Differentially correlated regulations in cancers** .......... 6
Figure S3: Cancer association of DC regulations .......... 7
**S4 Identification of STAT1-regulated functional modules** .......... 9
**S5 mRNA and miRNA expression profiles** .......... 11
Table S3: The number of samples for each cancer type. .......... 11
**S6 List of the drugs differentially regulated *STAT1* expression** .......... 11
**References** .......... 12

## S1 Descriptive statistics of the gene regulatory network structure

In this study, we collected transcription factor (TF) and microRNA (miRNA) regulations to construct global human gene regulatory networks (GRN) from predicted and experimentally validated data, respectively. This predicted GRN consists of 107 TFs, 1,851 mature miRNAs, 18,705 target genes, and 825,659 regulations among these molecules. The experimentally validated network consists of 10,046 regulations among 597 TFs, 497 miRNAs, and 2581 target genes. Detailed information regarding network structure for these two GRNs is depicted in Table S1.

Table S1: Number of regulations in the GRNs

Predicted

| Regulations | Transcription Factors | Mature miRNAs | Target Genes |
|---|---|---|---|
| Transcription Factors | 4,555 | 43,882 | 571,877 |
| Mature miRNAs | 3,029 | | 202,316 |

Experimentally validated

| Regulations | Transcription Factors | Mature miRNAs | Target Genes |
|---|---|---|---|
| Transcription Factors | 863 | 1,224 | 4,782 |
| Mature miRNAs | 761 | | 2,416 |

## S2 Reliability of the gene regulatory network

In the predicted GRN, we observed that in-degree, i.e. regulations to targets, showed scale-free distribution, but out-degree, i.e. regulations from regulators, did not (Figure S1A). To further confirm this, we investigated the degree distribution of the experimentally validated GRN. Interestingly, both the in-degree and out-degree of the experimentally validated GRN showed scale-free distribution (Figure S1B). These observations might uncover the high false positive rate of this predicted GRN. However, the experimentally validated GRN could be subject to publication bias, i.e. regulators studied more could possess more targets. Indeed, in the experimentally validated GRN, the TF and miRNA out-degree are both significantly and highly correlated with the number of publications (Spearman′s ρ, TF: 0.49, P < 2.2×10-16; miRNA: 0.68, P < 2.2×10-16); this positive correlation was observed only for miRNA in the predicted GRN (Spearman′s ρ, TF: 0.18, P = 0.06; miRNA: 0.36, P = 6.7×10-13) (Table S2). The information regarding the publications was obtained from National Center for Biotechnology Information (NCBI) database. These observations implied that the predicted GRN might possess a high false positive rate, but the experimentally validated GRN might be potentially biased by the number of publications.

Accordingly, we considered the expression correlation between regulators and target genes to filter out potential false positive regulations and publication bias. We incorporated the mRNA and miRNA expression profiles of seven cancer types from The Cancer Genome Atlas (TCGA). We then mapped the expression correlations of each regulation to the predicted GRN to contrast the correlated GRN for each cancer type. We observed that the highest out-degree of the correlated GRN can be controlled by around 1,000 when only the top 1%, 5%, or 10% highly correlated regulations are used for each cancer type (Figure S2). To note, the highest out-degree in the experimentally validated GRN is 648. Moreover, the averaged $R^2$ of out-degree distribution for the top 1%, 5%, and 10% highly correlated GRN was increased to around 0.5 (Normal: 1%: 0.48, 5%: 0.5, 10%: 0.45; Tumor: 1%: 0.51, 5%: 0.48, 10%: 0.49) (Figure S1). Notably, the $R^2$ of out-degree distribution for the predicted GRN is 0.17. Interestingly, the publication bias could also be reduced by incorporating expression correlations between regulators and targets the predicted GRN (Table S2). The above results suggested that the application of the expression correlations between regulators and targets may be able to reduce the false positive rate of the predicted GRN, control the out-degree distribution as scale-free, and

reduce publication bias.

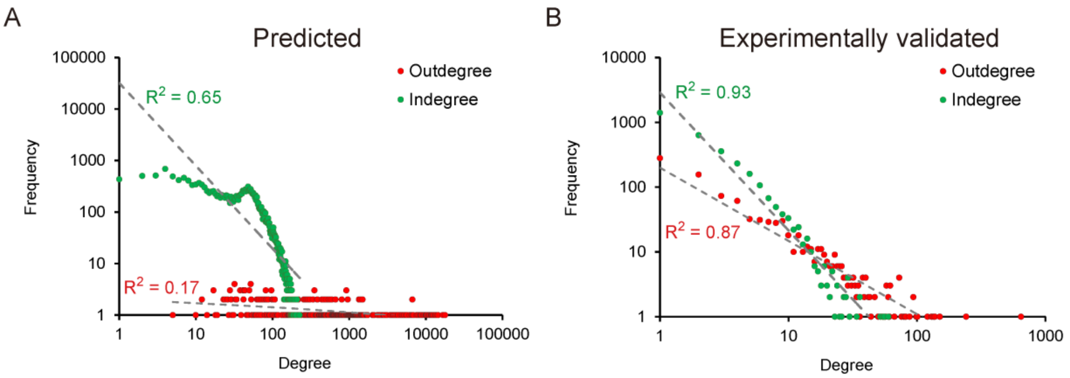


Figure S1: Regulation degree distribution of the GRNs

The degree distributions of (A) predicted (B) experimentally validated GRN. Out-degree: regulations from regulators; In-degree: regulations to targets.

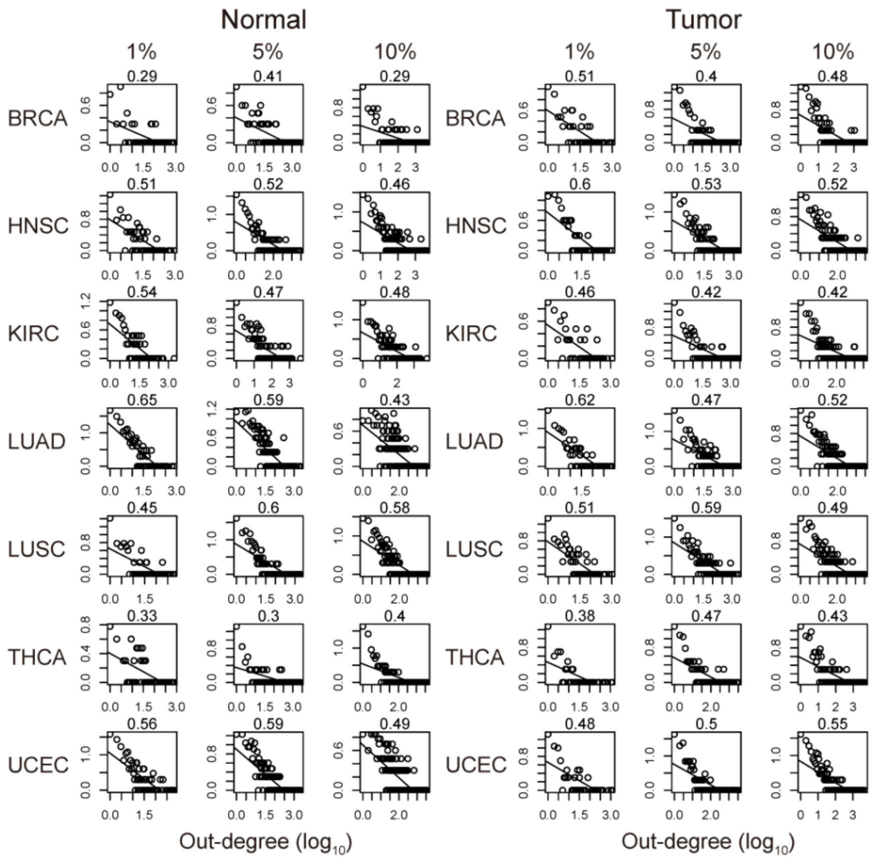


Figure S2: Out-degree distribution profile of correlated GRNs

The out-degree distribution profile of the top 1%, 5%, and 10% highly correlated GRN for each cancer type. The log10 frequency of regulators is shown as a function of the log10 out-degree of regulators. The $R^2$ of the out-degree distribution is labeled on the top of each sub-chart.

Table S2: Correlation between out-degree and the number of publications of regulators

| Tumor | | | | | |
|---|---|---|---|---|---|
| Gene | | | miRNA | | |
| Top(%) | SCC | *P*-value | Top(%) | SCC | *P*-value |
| Exp. validated | | | Exp. validated | | |
| 100% | 0.49 | < 2.2e-16 | 100% | 0.68 | < 2.2e-16 |
| Predicted | | | Predicted | | |
| 1% | -0.03 | 0.61 | 1% | 0.26 | 0.28 |
| 5% | 0.01 | 0.74 | 5% | 0.24 | 0.14 |
| 10% | 0.07 | 0.54 | 10% | 0.30 | 0.01 |
| 50% | 0.16 | 0.10 | 50% | 0.50 | < 0.01 |
| 100% | 0.18 | 0.06 | 100% | 0.36 | < 0.01 |

| Normal | | | | | |
|---|---|---|---|---|---|
| Gene | | | miRNA | | |
| Cancer | SCC | *P*-value | Cancer | SCC | *P*-value |
| Exp. validated | | | Exp. validated | | |
| 100% | 0.49 | < 2.2e-16 | 100% | 0.68 | < 2.2e-16 |
| Predicted | | | Predicted | | |
| 1% | 0.18 | 0.06 | 1% | 0.36 | < 0.01 |
| 5% | 0.07 | 0.47 | 5% | 0.16 | 0.33 |
| 10% | 0.06 | 0.52 | 10% | 0.20 | 0.14 |
| 50% | 0.09 | 0.40 | 50% | 0.23 | 0.01 |
| 100% | 0.17 | 0.08 | 100% | 0.41 | < 0.01 |

SCC: Average Spearman′s ρ across seven cancer types

*P*-value: Average *P*-value across seven cancer types

100%: The GRN without correlation filtering

## S3 Differentially correlated regulations in cancers

To probe the importance of differentially correlated (DC) regulations in cancers, we collected cancer-associated genes and miRNAs from public databases and literature (see Methods in the main text). The DC regulations were defined by the distance of Fisher transformed Spearman′s ρ between normal and tumor (see Methods in the main text). The cancer-associated regulations were those regulations formed by cancer-associated genes or cancer-associated miRNAs. We further categorized the cancer-associated regulations into three forms: 1) CN: only regulators are cancer-associated; 2) NC: only targets are cancer-associated; 3) CC: both regulators and targets are cancer-associated (C: cancer-associated, N: non-cancer-associated). Since there are two TFs as regulators in BiTT regulations, we combined CN and NC as CN, i.e. either one of the regulators of a BiTT is cancer-associated. On the other hand, because BiTM regulations possess two types of regulators, i.e. TF and miRNAs, we further specialized these three categories for BiTM as: 1) CN: only TFs are cancer-associated; 2) NC: only miRNAs are cancer-associated; 3): CC: both TFs and miRNAs are cancer-associated.

We observed that the cancer-associated regulations were significantly underrepresented in DC TFout regulations across seven cancer types (Figure S3A), even though the cancer-associated genes are significantly enriched in TFs (Figure S3B, left panel). This result implies that the cancer association significance of the DC TFout regulations might be diminished by the abundance of non-cancer-associated targets. Additionally, regulations composed of non-cancer-associated TFs and cancer-associated targets are significantly enriched in the DC TFout regulations (Figure S3A). Notably, the cancer-associated genes used in this study are required to be associated with cancer through mutation[1-3]. Therefore, this result proposes that these non-cancer-associated TFs with differential regulatory activity might be involved in cancer development through the regulation of cancer-associated targets rather than mutations.

Interestingly, BiTT regulations significantly overrepresented cancer-associated regulations across seven cancer types, especially for both of the TFs that are cancer-associated (Figure S3A). This observation might highlight the magnitude of regulatory FFL between two cancer-associated TFs across cancers. On the other hand, DC miRout regulations significantly overrepresented cancer-associated regulations across seven cancer types (Figure S3A). Additionally, the regulations formed by cancer-associated miRNAs (CN and CC) are significantly enriched in DC miRout regulations across seven cancer types. This result could be due to the significant enrichment of cancer-associated miRNAs involved in miRNA regulations (Figure S3B, right panel). This investigation also implies that miRNAs with distinct regulation activity might be involved in tumorigenesis even through targeting cancer-associated genes/TFs.

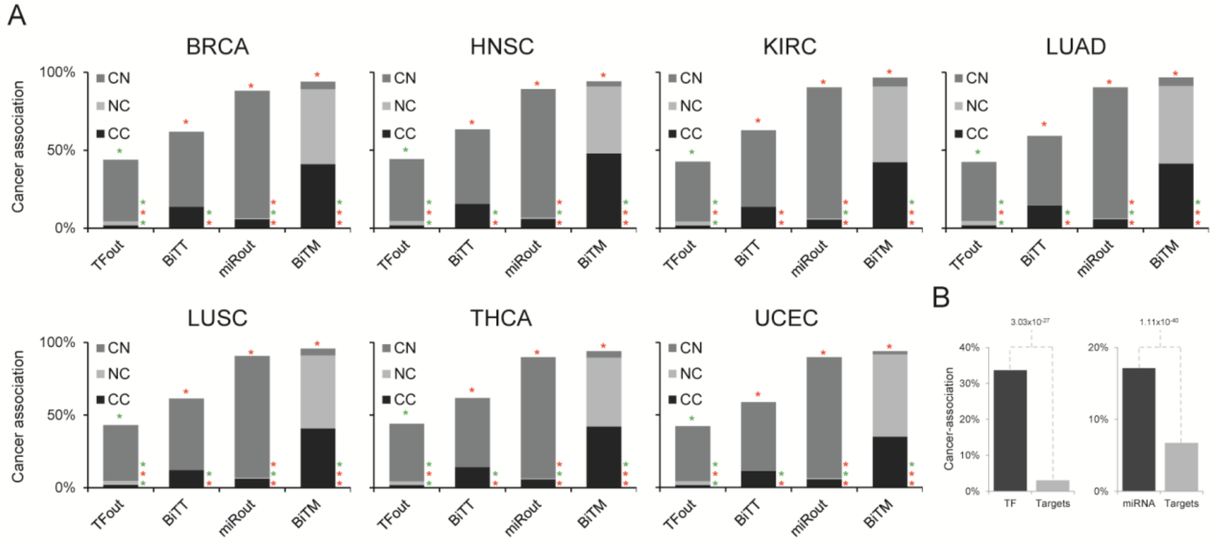


Figure S3: Cancer association of DC regulations

(A) The enrichment of cancer-associated regulations within four regulation types in the seven TCGA cancer types. For each cancer type, the proportion of cancer-associated regulations is shown for each regulation type. The asterisk at the top of each bar represents the significance of cancer-associated regulations with $P < 0.05$ derived from Fisher′s exact test. In addition, we

labeled the significance of the three sub-categorized cancer-associated regulations with $P < 0.05$ from Fisher's exact test at the bottom of each bar. The order of asterisks for the sub-categorized cancer-associated regulations is CN, NC, and CC from top to bottom (C: cancer-associated, N: non-cancer-associated). Red asterisk: significantly overrepresented. Green asterisk: significantly underrepresented. (B) The enrichment of cancer-associated TFs and cancer-associated miRNAs. The left panel shows the proportion of cancer-associated genes involved in TFs and the right one the proportion of cancer-associated miRNAs within miRNAs. The *P*-values are derived from Fisher's exact test.

## S4 Identification of STAT1-regulated functional modules

To discover the STAT1-regulated downstream functional modules, we collected STAT1 target genes that are significantly 1) positively co-expressed with *STAT1* and 2) up-regulated in tumor samples for each cancer type. Through these two conditions, we obtained those functional modules potentially activated by STAT1 in a tumor. The significantly positive co-expression was defined as the absolute standard score ≥ 2.5 (the corresponding significance is $P < 0.01$). The significant up-regulation in tumor is defined as edgeR $P < 0.05$, adjusted by the Benjamini and Hochberg multiple testing procedures[4].

Next, we performed functional enrichment analysis using Gene Ontology (GO)[5] annotations to determine the enriched functions in which these selected target genes are involved. Of note, we conducted the functional enrichment analysis in two ways, conventional and network-wise[6,7]. With the conventional way, the overrepresentation of selected STAT1 target genes defines the significance of STAT1-regulated functions. On the other hand, the network-wise enrichment analysis evaluates the significance of STAT1-regulated functions through the overrepresentation of functional protein-protein interactions (PPIs) among selected STAT1 targets. The PPIs were obtained from the Protein Interaction Network Analysis (PINA) v2[8,9]. Notably, the functional PPIs are PPIs formed by the two proteins involved in the same functions. Moreover, because we applied network-wise enrichment analysis, we further stipulated that the selected STAT1 target genes must be collected in the PPI network in the following analyses. The significance of each function is determined by the *P*-value produced from Hypergeometric test. For the conventional way, the hypergeometric distribution is:

$$P(X = k) = \frac{\binom{m}{k}\binom{N-m}{n-k}}{\binom{N}{n}}$$

where $X$ denotes the evaluated function. $N$ represents the number of GO annotated genes in the used expression profiles, as well as in PINA PPI network, while $m$ indicates that in the selected STAT1 target genes. $n$ represents the number of genes with the evaluated GO annotations in the used expression profiles as well as in PINA PPI network, while $k$ indicates that in the selected STAT1 target genes. Thus, this formula calculated the probability of the evaluated GO annotations that contains $k$ selected STAT1 target genes. For the network-wise way, we applied a modified hypergeometric distribution as below:

$$P_e(X = k_e) = \frac{\binom{m_e}{k_e}\binom{N_e - m_e}{n_e - k_e}}{\binom{N_e}{n_e}}$$

$e$ is the abbreviation of the functional PPIs. Each symbol represents the same meaning as the previous one in the conventional hypergeometric distribution, but the counting objects are changed from genes to functional PPIs. All the P-values are adjusted by the Benjamini and Hochberg multiple testing procedures to control the false discovery rate (FDR).

For each GO annotations, the two $P$-values produced by the conventional and network-wise method are further combined as a summarized $P$-value by Fisher′s method[10]. For each cancer type, a GO annotation is provided with a summarized $P$-value. We further combined these summarized $P$-values of a GO annotation as a combined $P$-value across the seven studied cancer types by Fisher′s method again. That is, we used the combined $P$-value to assess the enrichment consistency of the GO annotation across the studied seven cancer types. Furthermore, we utilized these combined $P$-values to rank the GO annotations in which the selected STAT1 target genes are involved. Finally, we considered the top 20 significant enriched GO annotations as potential STAT1-regulated downstream functions.

## S5 mRNA and miRNA expression profiles

The mRNA and miRNA expression profiles of seven cancer types in The Cancer Genome Atlas (TCGA) were investigated: breast cancer (BRCA), head and neck squamous cell carcinoma (HNSC), clear cell kidney carcinoma (KIRC), lung adenocarcinoma (LUAD), lung squamous cell carcinoma (LUSC), papillary thyroid carcinoma (THCA), and uterine corpus endometrial carcinoma (UCEC). The data was downloaded from TCGA on 10/02/2013. We used the normalized read counts inferred via RSEM (RNA-Seq by Expectation Maximization) algorithm[11] from RNA-Seq V2 as gene expressions. The RPM (Reads Per Million) values of miRNA-Seq data were used to represent the expression level of miRNA in the seven selected cancer types. Of note, to calculate Spearman′s ρ between miRNAs and target genes/TFs, only patient-matched samples between miRNA and mRNA expression profiles were used in this study. The numbers of samples for each cancer type are listed in Table S3.

Table S3: The number of samples for each cancer type.

| | BRCA | HNSC | KIRC | LUAD | LUSC | THCA | UCEC |
|---|---|---|---|---|---|---|---|
| Normal | 85 | 37 | 71 | 19 | 37 | 58 | 17 |
| Tumor | 654 | 264 | 208 | 400 | 325 | 485 | 118 |

## S6 List of the drugs differentially regulated *STAT1* expression

Supplementary file S1

## References


1 Vogelstein, B. *et al.* Cancer genome landscapes. *Science* **339**, 1546-1558 (2013).

2 Kandoth, C. *et al.* Mutational landscape and significance across 12 major cancer types. *Nature* **502**, 333-339 (2013).

3 Forbes, S. A. *et al.* COSMIC: mining complete cancer genomes in the Catalogue of Somatic Mutations in Cancer. *Nucleic Acids Res.* **39**, D945-950 (2011).

4 Benjamini, Y. & Hochberg, Y. Controlling the False Discovery Rate: A Practical and Powerful Approach to Multiple Testing. *J. R. Stat. Soc. Ser. B-Stat. Methodol.* **57**, 289-300 (1995).

5 Ashburner, M. *et al.* Gene ontology: tool for the unification of biology. The Gene Ontology Consortium. *Nature Genet.* **25**, 25-29 (2000).

6 Tseng, C. W., Lin, C. C., Chen, C. N., Huang, H. C. & Juan, H. F. Integrative network analysis reveals active microRNAs and their functions in gastric cancer. *BMC Syst. Biol.* **5**, 99 (2011).

7 Lin, C. C. *et al.* Dynamic functional modules in co-expressed protein interaction networks of dilated cardiomyopathy. *BMC Syst. Biol.* **4**, 138 (2010).

8 Cowley, M. J. *et al.* PINA v2.0: mining interactome modules. *Nucleic Acids Res.* **40**, D862-865 (2012).

9 Wu, J. *et al.* Integrated network analysis platform for protein-protein interactions. *Nat. Methods* **6**, 75-77 (2009).

10 Mosteller, F. & Fisher, R. A. Questions and Answers. *Am. Stat.* **2**, 30-31 (1948).

11 Li, B. & Dewey, C. N. RSEM: accurate transcript quantification from RNA-Seq data with or without a reference genome. *BMC Bioinformatics* **12**, 323 (2011).
